# Fixed-Bearing Versus Mobile-Bearing Prostheses in Total Ankle Arthroplasty: A Systematic Review and Meta-Analysis

**DOI:** 10.3390/jcm14176178

**Published:** 2025-09-01

**Authors:** Chiara Comisi, Domenico De Mauro, Tommaso Greco, Antonio Mascio, Virginia Cinelli, Giacomo Capece, Emidio Di Gialleonardo, Giulio Maccauro, Carlo Perisano

**Affiliations:** 1 Department of Orthopedics and Rheumatological Sciences, Fondazione Policlinico Universitario Agostino Gemelli IRCCS, 00136 Rome, Italy; chiara.comisi22@gmail.com (C.C.);; 2 Orthopaedics and Trauma Surgery Unit, Department of Ageing, Neurosciences, Head-Neck and Orthopaedics Sciences, Fondazione Policlinico Universitario Agostino Gemelli, IRCCS, 00136 Rome, Italy; 3 U.O.C. Orthopedics and Traumatology, University degli Studi di Napoli “Federico II” Via S. Pansini, 5, 80131 Naples, Italy; 4 Department of Life Sciences, Health, and Healthcare Professions, Link Campus University, 00165 Rome, Italy; 5 U.O.C. Orthopedics and Traumatology, Ospedale dei Pellegrini, 80134 Naples, Italy

**Keywords:** ankle arthritis, Total Ankle Replacement (TAR), mobile-bearing prosthesis, fixed-bearing prosthesis, implant failure, implant revision

## Abstract

**Background/Objectives:** Total ankle replacement (TAR) is considered an effective solution for end-stage ankle arthritis. New-generation implants have shown promising intermediate clinical outcomes and are available in two main designs: fixed-bearing and mobile-bearing prostheses. The aims of this study are to compare both prosthetic systems, focusing on (i) the revision rate for major complications, including conversion to arthrodesis, revision of components, and below-knee amputation; (ii) minor complications requiring additional surgery; and (iii) providing a comprehensive overview of total ankle replacement. **Methods:** A systematic review of the literature was conducted using the main databases. The inclusion criteria were patients aged 18 years or older and individuals who had undergone total ankle arthroplasty. Case reports, case series, original articles, and systematic reviews were excluded from the final selection. The pooled incidence of events was reported using odds ratios (ORs) with corresponding 95% confidence intervals (CIs). **Results:** A total of 33 studies, pooling data from 3652 observations and identifying 635 events, met the inclusion criteria. Relevant demographic and surgical data were systematically extracted and analyzed. A meta-analysis of comparable data revealed revision and failure risks for both prosthesis types. No statistically significant differences in complication rates were observed between fixed-bearing and mobile-bearing prostheses. **Conclusions:** Both fixed-bearing and mobile-bearing prostheses are viable options for treating ankle arthritis, demonstrating an intermediate risk of complications over short, medium, and long-term follow-ups.

## 1. Introduction

Ankle osteoarthritis (OA), characterized by degeneration of the tibiotalar joint, leads to significant pain and a decline in patient autonomy and quality of life [1]. While tibiotalar fusion remains the main solution for end-stage cases of ankle OA, particularly in young and adult patients, providing good long-term results, TAR is increasingly used as a viable alternative, demonstrating efficacy in enhancing the quality of life of patients [2]. Despite an associated high revision rate, contemporary implants have achieved promising intermediate clinical outcomes [3,4]. Over the past 15 years, the literature has increasingly supported the acceptance of TAR as a highly viable option to ankle arthrodesis, providing restoration of joint function, stability, and alignment, thereby improving ambulation and autonomy for patients [5].

Discussion about TAR is in a constant state of evolution. It is considered an ongoing challenge for orthopedic surgeons, particularly due to the prevalence of a high number of complications [6]. Third and fourth-generation implants are currently used worldwide, with medium- to long-term outcome studies emerging in the literature. Prosthetic evolution in TAR has also involved significant advancements in implant materials across generations. First-generation implants typically feature cemented designs made of stainless steel and polyethylene, which show high rates of loosening and wear. Second-generation implants introduce uncemented fixation with titanium alloy components and ultra-high-molecular-weight polyethylene (UHMWPE) inserts, aiming to improve osseointegration and reduce mechanical failure. Third-generation implants continue this trend, improving surface coatings such as hydroxyapatite or porous titanium to improve bone-implant integration. Finally, fourth-generation implants offer modular designs with improved biomaterials, including cobalt–chromium alloys for increased strength, refined UHMWPE to reduce wear, and titanium plasma spray or porous tantalum coatings to optimize fixation and durability. These advancements in materials have played a key role in reducing complication rates and improving implant longevity.

Modern prosthetic systems include both fixed-bearing (FB) and mobile-bearing (MB) design [7]. FB implants have shown a tendency for increased tibial implant loosening, attributed to stress forces at the bone–implant interface. Conversely, MB implants afford greater range of motion, with a heightened risk of impingement [8]. Notably, the literature lacks conclusive evidence delineating the comparative outcomes of these two implant types, and studies assessing their survivorship are unclear and ambiguous.

Therefore, this meta-analysis aims to (i) compare the risk of complications between fixed-bearing and mobile-bearing prosthesis systems, specifically evaluating minor, major, and total adverse events, and (ii) offer a comprehensive overview of the potential advantages and disadvantages associated with each implant type in terms of outcomes and overall effectiveness.

## 2. Materials and Methods

### 2.1. Search Strategy and Eligibility Criteria

According to the Preferred Reporting Items for Systematic Review and Meta-Analyses (PRISMA) guidelines [9], a systematic review of the literature was conducted up to December 2023. We focused on the examination of clinical outcomes and complications in patients undergoing either fixed-bearing or mobile-bearing total ankle arthroplasty. The search strategy encompassed three online databases: MEDLINE, Web of Science, and Scopus. The keywords used for the research were combined as follows: “TAA” or “TAR” or “total ankle arthroplasty” or “total ankle replacement” and “aseptic loosening” or “implant failure” or “mobilization” and “mobile bearing” or “fixed bearing” not “arthrodesis” and relative MeSH combinations.

To avoid overlap with other ongoing review studies, the protocol was registered online with the International Prospective Register of Systematic Reviews (PROSPERO) before submitting the review (ID CRD42024609409).

The inclusion criteria comprised (i) individuals aged 18 years or older and (ii) patients with a history of end-stage ankle osteoarthritis who had undergone total ankle replacement.

Case reports, expert opinions, previous systematic reviews, letters to the editor, similar meta-analyses, and studies with incomplete data were excluded from the study because they were unsuitable for data pooling.

### 2.2. Study Assessment and Data Extraction

Titles and abstracts of potential eligible studies were initially screened by two independent reviewers (DDM and TG). Subsequently, full texts were carefully read and selected, based on the inclusion criteria, by two independent reviewers (CC and AM). Any doubts or discrepancies were resolved by the senior author (CP). Relevant data were systematically extracted from each study, including participant demographics, sample size, hardware type, surgical approaches, radiological findings, as well as outcomes and complications.

A comparative statistical analysis was conducted for each group, involving the evaluation of the complication rate for each group, FB and MB ankle prosthesis systems. Data from each group were extracted in terms of (i) major events, including conversion to arthrodesis, revision of TAR, and below-knee amputation; (ii) minor events, including all minor complications that require a surgical re-intervention; and (iii) total events (minor and major events) for both FB and MB prosthesis systems.

The methodological quality of the studies incorporated into this meta-analysis was evaluated using the Methodological Index for Non-Randomized Studies (MINORS) score [10], which provides maximum scores of 16 and 22 for non-comparative and comparative studies, respectively. Two authors (GC and EDG) independently determined the MINORS score, and the final score was derived through consensus.

### 2.3. Statistical Analysis

The analysis employed the log odds ratio as the metric for outcomes. Considering the expected diversity among the included studies, a random-effects model was utilized for data fitting. The width of the heterogeneity (τ^2^) was estimated using the restricted maximum-likelihood estimator [11]. Moreover, the analysis presents the Q-test to assess heterogeneity and reports the I^2^ statistic [12]. If any level of heterogeneity is detected (τ^2^ > 0), a prediction interval for the true outcomes is also provided. Studentized residuals and Cook’s distances were utilized to evaluate whether individual studies could potentially act as outliers and/or exert influence within the model framework. Studies with a studentized residual exceeding the percentile of a standard normal distribution were deemed potential outliers. This classification employed a Bonferroni correction, utilizing a two-sided significance level of α = 0.05 for studies included in the meta-analysis. Studies with Cook’s distance exceeding the median plus six times the interquartile range of the Cook’s distances were identified as influential. Funnel plot asymmetry was evaluated using both the rank correlation test and the regression test, with the standard error of the observed outcomes serving as the predictor. This discrepancy between the two tests does not definitively indicate publication bias, but it raises the possibility of small-study effects or selective reporting. Given the heterogeneity in sample sizes and follow-up durations among the included studies, some degree of publication bias cannot be ruled out and should be considered when interpreting the pooled estimates. The pooled incidence of events was reported using odds ratios (ORs) along with corresponding 95% confidence intervals (CIs). Statistical analyses were conducted using SPSS version 29 (SPSS, Chicago, IL, USA). A significance level of *p* ≤ 0.05 was deemed significant. Additionally, the R software (version 4.3.1; R Foundation for Statistical Computing, Vienna, Austria) was used for meta-analytical plotting and visualization. Specifically, forest plots and funnel plots were generated using the “meta” and “metafor” packages. These tools allowed for enhanced control over heterogeneity modeling, outlier detection, and graphical output.

## 3. Results

### 3.1. Search and Selection Process

**Figure 1:**
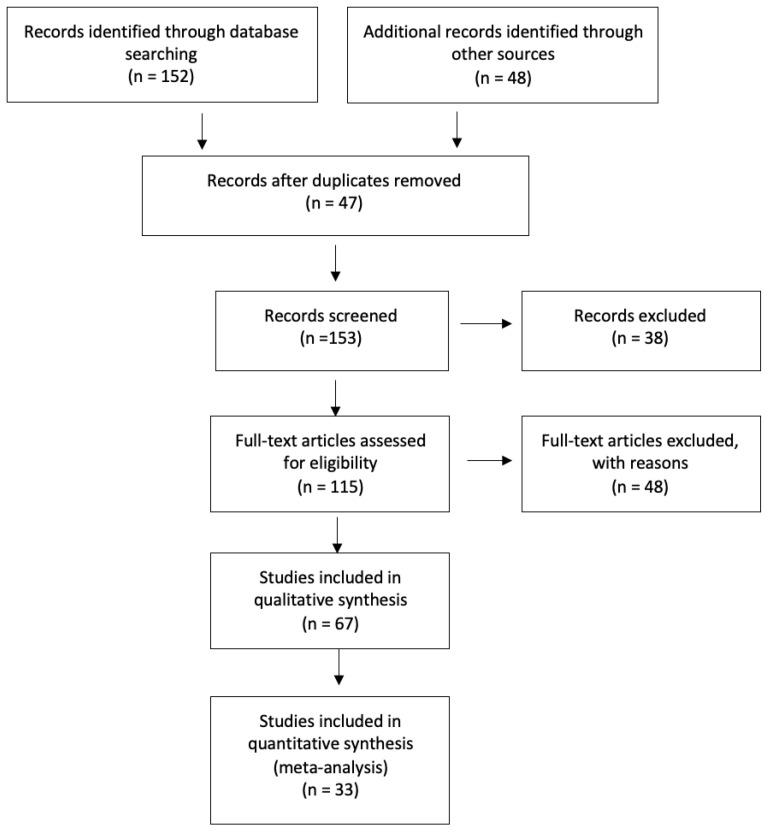
Study flowchart.

**Figure 2:**
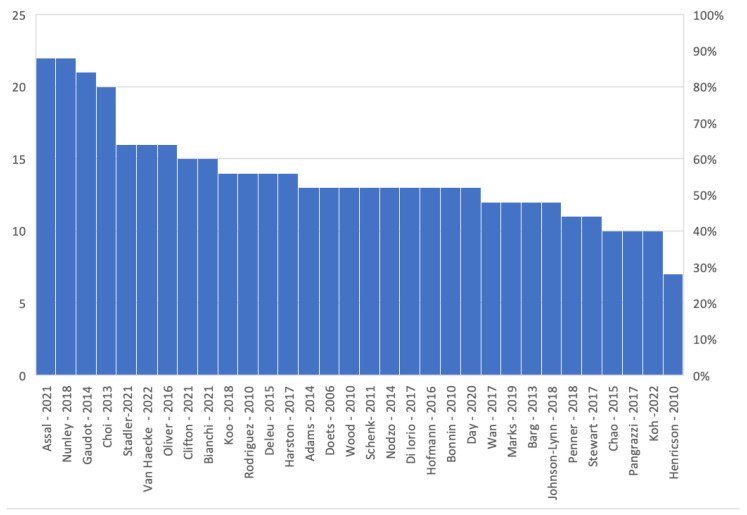
MINORS score.

The study flow chart is presented in Figure 1. A total of 200 articles were extrapolated from the initial literature search, with 47 duplicates studies subsequently removed. The remaining 153 papers underwent screening based on titles and abstracts. After excluding papers in languages other than English, the full texts of 67 articles were further assessed for eligibility. Through this full-text analysis, additional articles were included in the review, sourced from references in the full-text papers admitted for analysis. After excluding papers that did not meet the inclusion criteria, ultimately, 33 studies were included in this systematic review. The quality analysis of the included studies was assessed using the Methodological Index for Non-Randomized Studies (MINORS) score. Among the 20 comparative studies, the mean MINORS score was 20.0, with a range from 18 to 22. For the 13 non-comparative studies, the mean score was 13.4, ranging from 12 to 15. These findings indicate an overall moderate to high methodological quality across the selected studies from the literature. The results are shown in Figure 2.

### 3.2. Descriptive Data of Included Studies

Thirty-three studies were incorporated into the study, covering a timeframe spanning from 2006 to 2022. Altogether, 3616 patients and 3652 ankles that underwent TAR surgery were identified and included. The mean age ranged from 56 to 70 years, with a mean of 62.2 ± 5; the mean BMI was 27.7 ± 3.5; and 49% were male.

We included only patients who underwent TAR with third- and fourth-generation implants. We collected follow-up data and assessed complication rates, distinguishing between major and minor events. Major events included revision of one or both prosthetic components, conversion to arthrodesis, and below-knee amputations. Minor events included any other complications that necessitated reoperation, thereby indicating the need for additional surgical intervention.

All data were systematically collected and resumed in Table 1.

### 3.3. MB vs. FB: Complications

**Figure 3:**
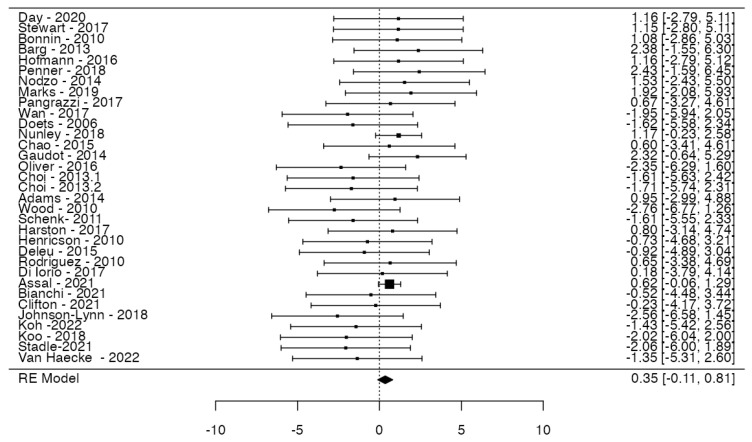
Forest plot of meta-analysis. Risks of total events in fixed-bearing prosthesis versus mobile-bearing ones. Data were extrapolated using random-effects models, and confidence intervals are represented by bars.

The comprehensive analysis encompassed 33 studies, pooling data from 3652 observations (involving patients with MB or FB), and identifying 635 events (complications). The average log odds ratio estimated through the random-effects model was 0.35 (95% CI: −0.11 to 0.81) (Figure 3). Consequently, no statistically significant difference was observed between the two groups (z = 1.498, *p* = 0.133). As per the Q-test, there was no significant heterogeneity in the true outcomes (Q = 23.84, *p* = 0.850, tau^2^ = 0.0000, I^2^ = 0.0000%).

**Figure 4:**
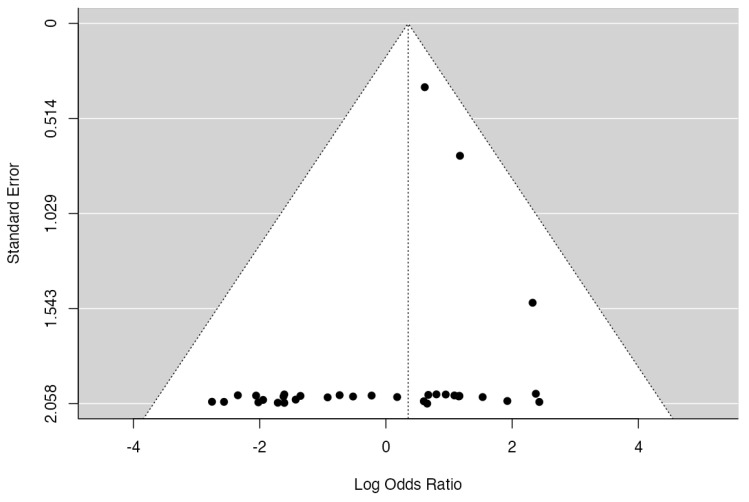
A funnel plot of the meta-analysis. None of the studies could be considered excessively influential. Neither the rank correlation nor the regression test revealed any asymmetry in the funnel plot.

Two studies (Nunley, 2018 [23]; Assal, 2021 [36]) had relatively large weights compared to the rest of the studies (so a weight at least three times as large as having equal weights across studies). An examination of the studentized residuals revealed that none of the studies had a value larger than ± 3.1718, and hence, there was no indication of outliers in the context of this model. According to the Cook’s distances, one study (Gaudot, 2014 [25]) could be considered to be overly influential. The rank correlation test indicated funnel plot asymmetry (*p* = 0.0184) but not the regression test (*p* = 0.0628) (Figure 4).

### 3.4. MB vs. FB: Major Events

**Figure 5:**
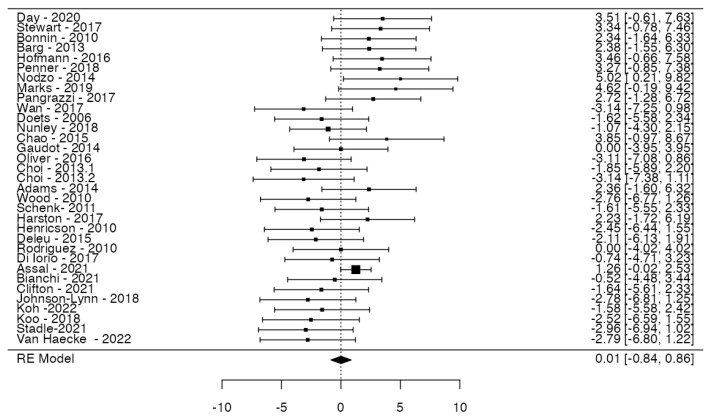
Forest plot of meta-analysis. Risks of major events in fixed-bearing prosthesis versus mobile-bearing ones. Data were extrapolated using random-effects models, and confidence intervals are represented by bars.

**Figure 6:**
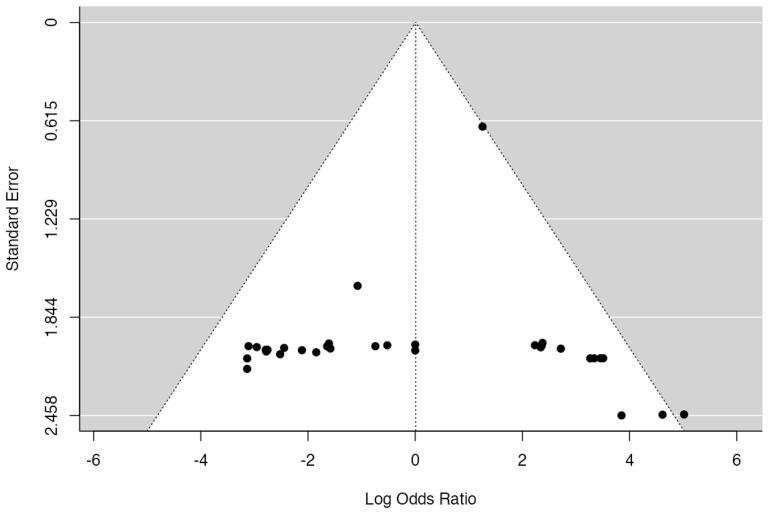
A funnel plot of the meta-analysis. None of the studies could be considered excessively influential. Neither the rank correlation nor the regression test revealed any asymmetry in the funnel plot.

A total of 33 studies were included in the analysis, pooling data from 3652 observations (involving patients with MB or FB) and identifying 292 major events. The estimated average log odds ratio based on the random-effects model was 0.01 (95% CI: −0.84 to 0.86) (Figure 5). Therefore, no statistically significant difference was observed between the two groups (z = 0.024, *p* = 0.980). According to the Q-test, the true outcomes appear to be heterogeneous (Q (32) = 53.2791, *p* = 0.0105, tau^2^ = 2.2650, I^2^ = 39.2838%). A 95% prediction interval for the true outcomes is given by −3.0605 to 3.0815. Hence, although the average outcome is estimated to be positive, in some studies, the true outcome may in fact be negative. An examination of the studentized residuals revealed that none of the studies had a value larger than ±3.1718, and hence, there was no indication of outliers in the context of this model. According to the Cook’s distances, none of the studies could be overly influential. Neither the rank correlation nor the regression test indicated any funnel plot asymmetry (*p* = 0.7702 and *p* = 0.8324, respectively) (Figure 6).

### 3.5. MB vs. FB: Minor Events

**Figure 7:**
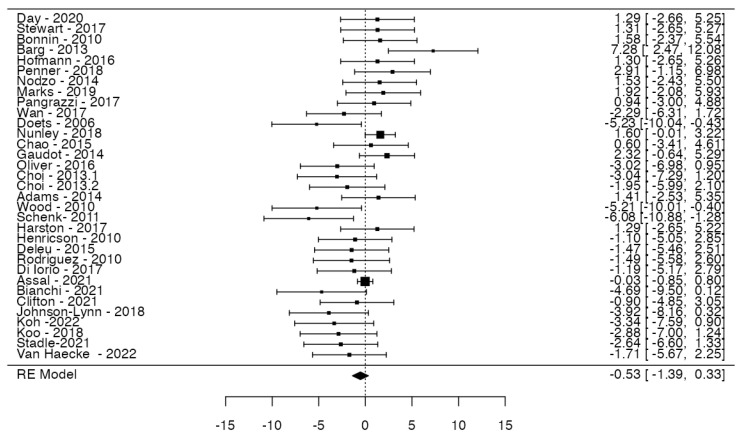
Forest plot of meta-analysis. Risks of minor events in fixed-bearing prosthesis versus mobile-bearing ones. Data were extrapolated using random-effects models, and confidence intervals are represented by bars.

**Figure 8:**
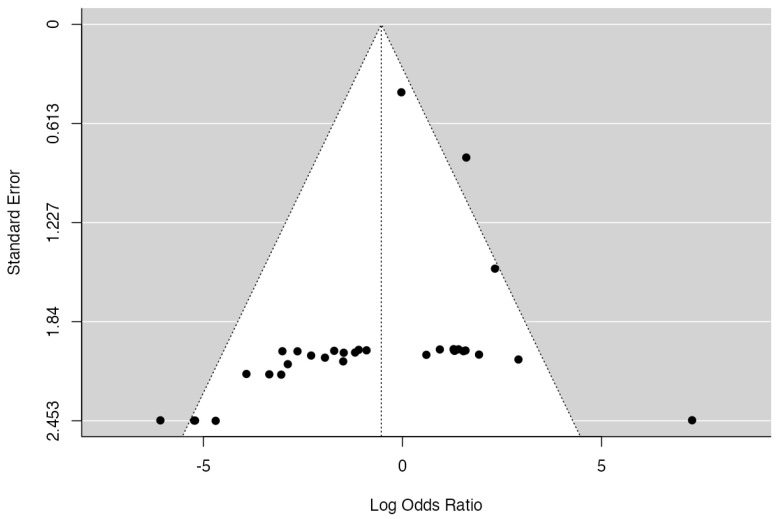
A funnel plot of the meta-analysis. None of the studies could be considered excessively influential. The rank correlation test indicated funnel plot asymmetry (*p* = 0.0001) but not the regression test.

A total of 33 studies were analyzed, pooling data from 3652 observations (involving patients with MB or FB) and identifying 339 minor events. The estimated average log odds ratio based on the random-effects model was −0.53 (95% CI: −1.39 to 0.33) (Figure 7). Consequently, no statistically significant difference was observed between the two groups (z = −1.203, *p* = 0.228). According to the Q-test, the true outcomes appear to exhibit heterogeneity (Q = 58.15, *p* = 0.003, tau^2^ = 2.5672, I^2^ = 48.7383%). An examination of the studentized residuals revealed that none of the studies had a value larger than ±3.1718, and hence, there was no indication of outliers in the context of this model. According to the Cook’s distances, none of the studies could be considered to be overly influential. The rank correlation test indicated funnel plot asymmetry (*p* = 0.0001) but not the regression test (*p* = 0.0702) (Figure 8).

## 4. Discussion

Total ankle replacement is a viable and definitive option for severe ankle osteoarthritis. The literature has shown that first- and second-generation prosthetic implants were associated with higher complication rates and frequent hardware failures. In fact, TAR remains a challenging procedure for orthopedic surgeons.

Newer-generation implants aim to address these issues and reduce the incidence of complications, particularly by improving design and material durability. In particular, modern prosthetic systems include both FB and MB designs.

In this study we conducted a systematic search in the literature, aiming to find all studies on patients who underwent TAR, to evaluate the mean differences between the two different types of study. With the growing number of patients affected by high grades of ankle osteoarthritis, the demand for total ankle replacement is expected to rise. Studies in the literature should be interpreted with caution. Current findings may be influenced by differences in surgeons’ experiences; TAR has been shown to involve a considerable learning curve. This factor could impact outcomes and complication rates, highlighting the importance of considering surgeon experience when assessing TAR results [8,44]. Despite this, only a few studies in the literature, and notably one meta-analysis, have focused on adverse event rates following TAR. We believe this is a highly relevant area of study as it provides valuable insights for surgeons. Our findings reveal no statistically significant differences in terms of total, minor, or major adverse events between MB and FB prosthetic designs. Unlike M. González-Alonso et al. [7], who included second-, third-, and fourth-generation implants in their meta-analysis, we restricted our study to third- and fourth-generation implants. Survival rates for fixed-bearing and mobile-bearing TAR were 94% and 89% (95% CI [0.86; 0.92]), respectively, indicating that no significant difference was observed between the two groups in the current literature. Nevertheless, our results align with theirs, demonstrating no statistically significant differences between MB and FB prostheses.

Moreover, most of the studies included in our analysis have a long follow-up period. Notably, Bonnin, Bianchi, Di Iorio, and colleagues conducted studies with ten-year follow-ups, reporting implant survival rates of 65%, 66%, and 68%, respectively [15,35,37].

Currently, there are limited studies with long-term follow-ups on fourth-generation TAR implants, while third-generation prostheses are more thoroughly documented. In our review, we included a study by Penner et al. [6], who reported outcomes for a fourth-generation TAR, the Infinity total ankle system. Their findings indicate that early clinical and radiographic outcomes with this implant are promising, comparing favorably to those of both fixed- and mobile-bearing third-generation TAR designs, even in cases with deformities and greater complexity.

Adverse events and failure rates remain the most concerning complications following TAR. The literature reports several complications, including surgical site infections (SSIs), cystic lytic lesions, intraoperative fractures, polyethylene fractures, impingement, neurovascular injuries, component subsidence, non-union, and painful hardware [45]. An interesting radiologic evaluation was conducted by Van Haecke et al. [43], who used both preoperative and postoperative CT scans to enhance cyst analysis. Additionally, they utilized pre- and postoperative dynamic X-rays to assess implant motion: radiologic measurements included angles, the talar component slope, and the hindfoot axis. At the last follow-up, no cases of implant subsidence were observed. However, the most serious complications are implant failure, deep infections, and aseptic loosening. In these severe cases, final treatment options include revision of one or both components, conversion to arthrodesis, or, in the most critical cases, below-knee amputation. The study was designed to obtain a clear statement regarding the comparison of the risk of complications between fixed-bearing and mobile-bearing prosthesis systems, specifically evaluating minor, major, and total adverse events. Our findings indicate a slightly higher, though not statistically significant, prevalence of major adverse events in MB prosthetic implants. Furthermore, our data indicate a slightly higher, though not statistically significant, prevalence of minor events in fixed-bearing prosthetic implants. Findings for total events were overall similar between the two types of implants. Consequently, both mobile-bearing and fixed-bearing ankle prostheses appear to be viable options for the treatment of ankle arthritis, each presenting an intermediate risk of complications in the short-, medium-, and long-term follow-up periods.

Therefore, the primary strength of this study lies in its significant contribution to the literature, providing valuable meta-analytic data that can guide surgeons in clinical practice, and offering a useful resource for the scientific community. The inclusion of studies with long follow-up periods further strengthens the robustness of our findings. The study also has several limitations, including the exclusion of concomitant surgical procedures performed alongside total ankle replacement. Additionally, minor non-surgical complications, which could still elevate the risk of revision, were not included.

However, these results should not be interpreted as proof of equivalence between the two implant types. Rather, they highlight the need for caution in drawing firm conclusions, particularly in the absence of high-level evidence and long-term prospective studies. Consequently, heterogeneity among the included studies was high. Although we statistically accounted for it using a random-effects model, subtle clinical differences between the implant types may exist and could become evident in more homogeneous or better-powered analyses.

Several limitations should be acknowledged. First, the level of evidence of the included studies is generally low, with the majority being retrospective cohort studies or case series. To mitigate this, we included only studies with mid- to long-term follow-up. Second, the definition of implant failure is not standardized across studies, limiting the comparability of the reported outcomes.

These limitations underscore the necessity for future research, particularly well-designed randomized controlled trials with standardized outcome definitions and extended follow-up. Such studies will be essential to better understand the comparative effectiveness and safety of fixed- versus mobile-bearing prostheses, ultimately improving clinical decision-making and patient care in total ankle arthroplasty.

## 5. Conclusions

Based on the data revealed in this meta-analysis, while significant advancements have been made in the field of TAR, it remains a challenging procedure for surgeons, with potential severe adverse outcomes. Both fixed-bearing and mobile-bearing TAR have demonstrated acceptable survival rates without significant differences between them. While no statistically significant differences were observed between the two groups, pooled complication rates revealed total event rates of 16.1% for FB implants and 18.8% for MB implants. Major complication rates were 6.8% for FB and 9.2% for MB, while minor complication rates were 9.2% and 9.6%, respectively. The current lack of extensive long-term follow-up studies and high-level evidence complicates the ability to accurately predict the survivorship of implants. These complexities underscore the necessity for further research to optimize surgical techniques, refine patient selection criteria, and improve postoperative management. Continued investigation into these aspects will be crucial in enhancing the safety and effectiveness of TAR, ultimately contributing to better patient outcomes and satisfaction.

## Figures and Tables

**Table 1:** Demographic characteristics and specific data of included studies. NR (not reported); FU (follow-up); FB (fixed-bearing); MB (mobile-bearing).

First Author—Year Published	Study Type	N. Patients	N Ankles	Sex (Male)	Age (Mean)	BMI (kg/m^2^)	FU (Months) (Mean)	Hardware Type	Insert Type	Reoperation	Revision	Revision of 1 or Both Components	Arthrodesis	Underwent Below-the-Knee Amputations
Day, 2020 [13]	Retrospective study	82	85	34	63.5	28.1	85.2	Salto Talaris	FB	18	2	2	0	0
Stewart, 2017 [14]	Retrospective study	72	72	22	61.9	29.6	81.1	Salto Talaris	FB	17	2	2	0	0
Bonnin, 2010 [15]	Retrospective study	96	96	36	56	24	106.8	Salto	FB	16	8	2	6	0
Barg, 2013 [16]	Retrospective study	684	722	387	61.1	26.9	75.6	HINTEGRA	FB	0	61	54	7	0
Hofmann, 2016 [17]	Retrospective study	78	81	32	64.3	29.4	64.2	Salto Talaris	FB	17	2	2	0	0
Penner, 2018 [6]	Retrospective study	67	67	37	61.5	29.8	35.4	Infinity	FB	6	2	2	0	0
Nodzo, 2014 [18]	Retrospective study	74	75	33	60.6	31	43	Salto Talaris	FB	13	0	0	0	0
Marks, 2019 [19]	Retrospective study	46	50	25	65.3	29.4	58.8	Salto Talaris	FB	6	0	0	0	0
Pangrazzi, 2017 [20]	Retrospective study	96	104	48	65	29.0	46	Salto Talaris	FB	29	6	5	1	0
Wan, 2017 [21]	Retrospective study	59	59	31	64.1	26.1	35.9	Salto	MB	5	2	0	2	0
Doets, 2006 [22]	Prospective study	76	93	63	57.6	NR	91.2	LCS	MB	0	15	2	13	0
Nunley, 2018 [23]	Prospective randomized trial	84	84	31	NR	NR	54	STAR; Salto Talaris	MB; FB	3 (FB); 8 (MB)	1 (FB)	1 (fixed)	0	0
Chao, 2015 [24]	Retrospective study	23	23	6	68.6	28.4	36	Salto	FB	8	1	0	1	0
Gaudot, 2014 [25]	Retrospective study	66	66	18; 19	64	NR	24; 23	Salto; Salto Talaris	MB; FB	4 (MB)	1 (FB)	0	1 (FB)	0
Oliver, 2016 [26]	Retrospective study	245	245	131	65.5	NR	38.9	Salto Talaris	MB	11	10	2	8	0
Choi, 2013 [27]	Retrospective study	102	67	63	21;20	NR	53; 34	Hintegra; Mobility	MB	1; 4	4; 1	4 (Hintegra)	1 (Mobility)	0
Adams, 2014 [28]	Retrospective study	194	194	135	64	NR	44.4	INBONE	FB	28	12	6	6	0
Wood, 2010 [29]	Prospective study	94	91	53	60	NR	43	Mobility	MB	0	5	3	2	0
Schenk, 2011 [30]	Prospective study	218	218	NR	56.8	NR	29	Salto	MB	0	36	24	12	0
Harston, 2017 [31]	Retrospective study	142	149	93	63.2	29.3	70.8	INBONE I	FB	32	14	9	4	1
Henricson, 2010 [32]	Retrospective study	93	93	35	NR	NR	42	AES	MB	23	7	3	4	0
Deleu, 2015 [33]	Retrospective study	50	50	25	54.9	27.1	45	Hintegra	MB	9	5	2	3	0
Rodriguez, 2010 [34]	Retrospective study	18	18	8	57.6	NR	39.4	AES	MB	3	0	0	0	0
Di Iorio, 2017 [35]	Prospective study	44	50	27	56	28	120	AES	MB	0	20	0	20	0
Assal, 2021 [36]	Retrospective study	302	302	63; 87	68; 62	27.6; 28	36	Salto; Salto Talaris	FB	22 MB, 11 FB	22 MB, 5 FB	6 MB, 1 FB	7 MB, 2 FB	0
Bianchi, 2021 [37]	Retrospective study	52	54	NR	NR	NR	120	BOX	MB	0	20	9	11	0
Clifton, 2021 [38]	Retrospective study	70	70	54	69	NR	76	Hintegra	MB	9	11	8	3	0
Johnson, Lynn, 2018 [39]	Prospective study	76	76	52	63	28.5	60	Mobility	MB	1	4	4	0	0
Koh, 2022 [40]	Retrospective study	43	43	19	60	26.7	96	Mobility	MB	1	7	4	3	0
Koo, 2018 [41]	Retrospective study	42	46	33	70	NR	60	Salto	MB	2	3	2	1	0
Stadler, 2021 [42]	Retrospective study	169	171	87	60.5	NR	86.4	Salto	MB	11	8	3	5	0
Van Haecke, 2022 [43]	Retrospective study	94	94	57	62.4	27.5	81.4	HINTEGRA	MB	14	5	3	2	0

## Data Availability

Not applicable.
